# Cardiovascular Safety and Benefits of Semaglutide in Patients With Type 2 Diabetes: Findings From SUSTAIN 6 and PIONEER 6

**DOI:** 10.3389/fendo.2021.645566

**Published:** 2021-03-29

**Authors:** Michael A. Nauck, Daniel R. Quast

**Affiliations:** Diabetes Division, Katholisches Kinikum Bochum, St. Josef-Hospital, Ruhr-University of Bochum, Bochum, Germany

**Keywords:** glucagon-like peptide 1 receptor agonist, type 2 diabetes, cardiovascular, safety, semaglutide

## Abstract

To exclude an excess risk of cardiovascular (CV) events, CV outcomes trials (CVOTs) have assessed the effects of new glucose-lowering therapies, including glucagon-like peptide-1 receptor agonists (GLP-1RAs), in patients with type 2 diabetes and established CV disease or CV risk factors. The CV safety of semaglutide vs. placebo, when added to standard care, was evaluated in the SUSTAIN 6 trial for the formulation administered once-weekly subcutaneously and in PIONEER 6 for the new once-daily oral formulation. In SUSTAIN 6 and PIONEER 6, both powered to demonstrate noninferiority (upper 95% confidence interval [CI] of the hazard ratio [HR] <1.8), there were fewer first major adverse CV events with semaglutide vs. placebo, with HRs of 0.74 (95% CI 0.58–0.95) and 0.79 (0.57–1.11), respectively. In SUSTAIN 6, the results were significant for noninferiority and superiority, although the latter was not prespecified. Surprisingly, CV and all-cause mortality were significantly reduced by oral semaglutide in PIONEER 6. The ongoing SOUL CVOT will further inform about CV outcomes with oral semaglutide vs. placebo (NCT03914326). Findings from SUSTAIN 6 and PIONEER 6 fall within the spectrum reported with other GLP-1RA CVOTs: noninferiority vs. placebo for major CV events was seen with lixisenatide and exenatide extended-release, while superiority was demonstrated with liraglutide, albiglutide, and dulaglutide. Beneficial outcomes have been recognized in international guidelines, which recommend subcutaneous liraglutide, semaglutide, and dulaglutide to reduce the risk of CV events in high-risk patients. Both indirect mechanisms *via* risk factor modification and direct effects *via* GLP-1 receptors in the CV system have been proposed to be responsible for CV event reductions. The exact mechanism(s) remains to be characterized, but appears to be mainly linked to anti-atherosclerotic effects. Further research is needed to elucidate the relevant mechanisms for CV benefits of GLP-1RAs.

## Introduction

Independent of other conventional risk factors, diabetes confers an approximately two-fold increased risk for cardiovascular (CV) disease (CVD) compared with individuals without diabetes (1). The elevated risk of CVD begins at fasting glucose levels below the cut-off point for diabetes (<7 mmol/L [126 mg/dL]) and increases with increasing glucose levels (1). Approximately one-third of all individuals with type 2 diabetes (T2D) are or will be affected by CVD and it is a major cause of mortality, accounting for around half of all deaths (2).

Previously, concern was raised about the CV safety of glucose-lowering treatments for T2D, leading the US Food and Drug Administration to issue guidance for industry to ensure that evaluation of new therapies excluded an excess in CV risk (3). Since then, several CV outcomes trials (CVOTs) have been conducted, either with approved medications or with agents in development as part of the regulatory process.

In large prospective, randomized clinical trials, the CV safety of glucagon-like peptide-1 (GLP-1) receptor agonists (GLP-1RAs) was studied in comparison with placebo (both on a background of standard care); either neutral effects or reductions in CV events have been reported (4–10). The present article will review the Semaglutide Unabated Sustainability in Treatment of Type 2 Diabetes (SUSTAIN) 6 trial and the Peptide InnOvatioN for Early diabEtes tReatment (PIONEER) 6 trial, which were designed to evaluate the CV safety of subcutaneous and oral semaglutide, respectively (6, 10). For reference, the effects of other GLP-1RAs on CV events will also be compared. Potential mechanisms explaining reductions in CV events with GLP-1RAs in general, and with semaglutide in particular, will be discussed.

## SUSTAIN 6 – Establishing the Cardiovascular Safety of Subcutaneous Semaglutide

The preapproval SUSTAIN 6 trial aimed to prove noninferiority of subcutaneous semaglutide as compared with placebo for the primary endpoint of time to the first occurrence of a major adverse CV event (MACE), which was defined as death from CV causes, nonfatal myocardial infarction (MI), or nonfatal stroke (6). The trial was designed such that noninferiority of subcutaneous semaglutide to placebo was confirmed if the upper bound of the 95% confidence interval (CI) of the hazard ratio (HR) for the primary endpoint was below 1.8 (ruling out an 80% elevation in risk with an error margin of 5%). The trial was both time-driven (minimum duration of 104 weeks) and event-driven, with at least 122 primary outcome events needed for sufficient power to determine noninferiority.

SUSTAIN 6 was conducted in patients with T2D with HbA_1c_ >7% and at high risk of CV events, defined as: i) aged ≥50 years with established CVD (previous CV, cerebrovascular, or peripheral vascular disease), chronic heart failure (HF) (New York Heart Association [NYHA] class II or III), or chronic kidney disease (CKD) stage ≥3; or ii) aged ≥60 years with at least one CV risk factor (persistent microalbuminuria or proteinuria, hypertension with left ventricular hypertrophy, left ventricular systolic or diastolic dysfunction, or an ankle–brachial index <0.9) (6).

Patients were randomized (1:1:1:1) to receive either 0.5 mg or 1.0 mg of once-weekly subcutaneous semaglutide or volume-matched placebo in addition to their standard care for 104 weeks (6). A fixed-dose escalation procedure was used to minimize the gastrointestinal adverse events seen with GLP-1RAs, with a starting dose of subcutaneous semaglutide of 0.25 mg for 4 weeks, which was escalated to 0.5 mg for 4 weeks until the maintenance dose (0.5 mg or 1.0 mg) was reached.

Of the 3,297 patients enrolled, 83.0% were aged ≥50 years and had established CVD or CKD: 58.8% had established CVD without CKD (**Table 1**), 10.7% had CKD only, and 13.4% had both CVD and CKD (6). In total, 17% of the participants were aged ≥60 years and had at least one CV risk factor. The overall mean duration of T2D was 13.9 years, and the mean glycated hemoglobin (HbA_1c_) level was 8.7%. Background standard-of-care at baseline included metformin for 73% of patients, insulin for 58%, and sulfonylureas for 43%. Most patients were receiving antihypertensive medication (93%), lipid-lowering therapy (76%), and antithrombotic/antiplatelet drugs (76%).

**Table 1:** Baseline characteristics of patients in the SUSTAIN 6 and PIONEER 6 trials [Marso (6); Husain (10)].

Trial	SUSTAIN 6[Marso 2016a]	PIONEER 6[Husain 2019]
Comparison	Once-weekly subcutaneous semaglutide 0.5/1.0 mg vs. placebo	Once-daily oral semaglutide 14 mg vs. placebo
*N*	3,297	3,183
Age, y	65 ± 7	66 ± 7
Female sex, %	39.3	31.6
Diabetes duration, y	13.9 ± 8.1	14.9 ± 8.5
HbA_1c_, %	8.7 ± 1.5	8.2 ± 1.6
Body weight, kg	92.1 ± 20.6	90.9 ± 21.2
Body mass index, kg/m²	32.8 ± 6.2	32.3 ± 6.5
Age ≥50 years and presence of CVD and/or CKD*, %	83.0	84.7
Age ≥60 years and presence of CV risk factors only, %	17.0	15.3
Established CVD without CKD, %	58.8	NA
CKD without CVD, %	10.7	NA
Established CVD with CKD, %	13.4	NA
Prior myocardial infarction, %	32.5	36.1
Prior heart failure (NYHA class II or III), %	23.6	12.2
Prior moderate renal impairment, %	25.2	28.2

Mean values ± standard deviation unless otherwise stated.

*CKD was taken as an equivalent to existing CVD.

CKD, chronic kidney disease; CV, cardiovascular; CVD, cardiovascular disease; HbA_1c_, glycated hemoglobin; NA, not available; NYHA, New York Heart Association; y, years.

**Figure 1:**
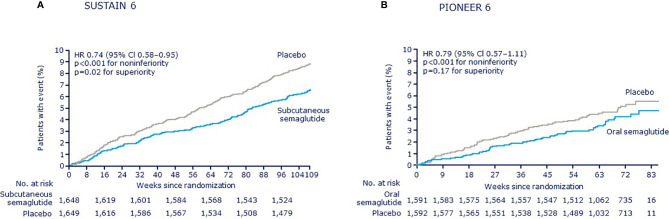
Kaplan–Meier plots of the primary outcome (a composite of cardiovascular death, nonfatal myocardial infarction, or nonfatal stroke) in SUSTAIN 6 and PIONEER 6 (11). **(A)** SUSTAIN 6 **(B)** PIONEER 6; CI, confidence interval; HR, hazard ratio. **(A)** From Marso SP, et al. Semaglutide and cardiovascular outcomes in patients with type 2 diabetes. N Engl J Med (2016) 375:1834-44. Copyright ^©^ (2016) Massachusetts Medical Society. Reprinted with permission from Massachusetts Medical Society. **(B)** From Husain M, et al. Oral semaglutide and cardiovascular outcomes in patients with type 2 diabetes. N Engl J Med (2019) 381:841-51. Copyright ^©^ (2019) Massachusetts Medical Society. Reprinted with permission from Massachusetts Medical Society.

Over 104 weeks, a first MACE was reported in 6.6% of patients who received subcutaneous semaglutide (both semaglutide doses combined) vs. 8.9% with placebo (both placebo groups combined), with a HR of 0.74 and a 95% CI of 0.58–0.95, confirming noninferiority to placebo (*p* < 0.001) (**Figure 1**) (6). As a preapproval trial, the main aim of SUSTAIN 6 was to confirm CV safety. As such, the trial was not powered to demonstrate superiority and such testing was not prespecified. However, the treatment effect of subcutaneous semaglutide and the accrual of more events than estimated resulted in a nominally significantly lower risk of MACE among patients receiving subcutaneous semaglutide (*p* = 0.02), as assessed *post-hoc*.

When the individual components of the primary endpoint were analyzed, nonfatal MI occurred in 2.9% of patients receiving subcutaneous semaglutide and in 3.9% of those receiving placebo (HR 0.74; 95% CI 0.51–1.08; *p* = 0.12), while nonfatal stroke occurred in 1.6% and 2.7%, respectively (HR 0.61; 95% CI 0.38–0.99; *p* = 0.04). Rates of death from CV causes were similar for subcutaneous semaglutide and placebo (2.7% vs. 2.8%, respectively; HR 0.98; 95% CI 0.65–1.48; *p* = 0.92), as was death from any cause (3.8% vs. 3.6%, respectively; HR 1.05; 95% CI 0.74–1.50; *p* = 0.79). An expanded composite endpoint of MACE plus revascularization (coronary or peripheral), and hospitalization for unstable angina or HF, occurred in 12.1% of patients receiving subcutaneous semaglutide and in 16.0% of patients receiving placebo (HR 0.74; 95% CI 0.62–0.89; *p* = 0.002). Thus, among patients with T2D at high CV risk, noninferiority was confirmed, and in a *post-hoc* non-prespecified analysis, the rate of MACE was shown to be significantly lower in those receiving subcutaneous semaglutide than in those receiving placebo.

## SELECT – Assessing the Cardiovascular Benefit of Subcutaneous Semaglutide in Patients With Overweight or Obesity

Following the confirmation that subcutaneous semaglutide is associated with CV safety and preliminarily, even some evidence of benefit, a definitive CVOT is ongoing to assess the effects of subcutaneous semaglutide on CV events in patients at high CV risk who are overweight or obese (NCT03574597) (12). In the ongoing Semaglutide Effects on Cardiovascular Outcomes in People with Overweight or Obesity (SELECT) trial, approximately 17,500 people with pre-existing CVD and with overweight or obesity (body mass index ≥27 kg/m^2^) but without diabetes will receive either subcutaneous semaglutide (up to 2.4 mg) or placebo in addition to standard care for up to 5 years. This will be the first clinical trial to assess the superiority of a GLP-1 RA versus placebo for reduction of CV events in patients with established CVD and overweight or obesity but without established T2D. By excluding patients with T2D, the aim is to reduce the extent to which improved glycemic control is the driver of improved CV outcomes. This could potentially show the benefit or early intervention in patients with normoglycemia or pre-diabetes even before the development of T2D and a positive outcome could indicate a new approach to CV risk reduction in obese or overweight patients. The primary endpoint of the SELECT trial is time to the first occurrence of MACE and the trial is powered to show superiority (semaglutide vs. placebo). Secondary endpoints include several composite CV endpoints, individual components, all-cause mortality, glycemic parameters, and changes in weight-related patient-reported outcomes.

## PIONEER 6 – Establishing the Cardiovascular Safety of Oral Semaglutide

Similar to SUSTAIN 6, PIONEER 6 aimed to establish the CV safety of oral semaglutide before regulatory approval and was not powered to prove superiority and, thus, CV benefit (10). As with SUSTAIN 6, PIONEER 6 was designed to establish noninferiority by ruling out an 80% excess in CV risk with oral semaglutide for noninferiority relative to placebo for an identical MACE primary outcome, but was driven by events only (at least 122 events needed to be accrued) and there was no minimum duration.

The eligibility criteria were almost identical to those of the SUSTAIN trial except there was no requirement for HbA_1c_>7% in PIONEER 6 and different restrictions on permitted background glucose-lowering medication. PIONEER 6, but not SUSTAIN 6, excluded patients with estimated glomerular filtration rate (eGFR) <30 mL/min/1.73 m^2^ and patients with proliferative retinopathy or maculopathy requiring acute treatment.

In PIONEER 6, patients were randomized (1:1) to receive once-daily oral semaglutide or placebo, both in addition to standard care. Slow dose escalation was used to minimize adverse events. Oral semaglutide was initiated at 3 mg and dose-escalated every 4 weeks, to 7 mg and then 14 mg. Once the maximum 14 mg daily dose was reached, patients remained at this dose unless a reduction was warranted due to adverse events.

Of the 3,183 patients enrolled in PIONEER 6, 85% were aged ≥50 years with established CVD and/or CKD and 15% were aged ≥60 years with CV risk factors only (**Table 1**). In total, 26% had moderate renal impairment (30 to <60 mL/min/1.73 m^2^). The mean HbA_1c_ level was 8.2%, which is lower than in SUSTAIN 6, perhaps reflecting the lack of a HbA_1c_ threshold in the inclusion criteria for PIONEER 6. The overall mean duration of T2D was comparable with SUSTAIN 6 at 14.9 years. Background standard-of-care at baseline included metformin for 77% of patients, insulin for 61%, and sulfonylureas for 32%. Compared with SUSTAIN 6, PIONEER 6 included a greater proportion of patients receiving sodium-glucose co-transporter-2 inhibitors (SGLT2is; 10% vs. <1%), reflecting the increased use of this drug class at the time of this trial.

In PIONEER 6, over a median follow-up of 15.9 months, the composite primary endpoint of MACE was reported in 3.8% of patients in the oral semaglutide group vs. 4.8% in the placebo group, with a HR of 0.79 and a 95% CI of 0.57–1.11, confirming noninferiority of oral semaglutide to placebo (*p* < 0.001) (**Figure 1**) (10). PIONEER 6 was not powered to assess superiority and a significant difference for the obvious trend between treatment groups was not detected (*p* = 0.17). When the individual MACE components were analyzed, a nominally statistically significant reduction in the risk of death from CV causes was observed (0.9% vs. 1.9%; HR 0.49; 95% CI 0.27–0.92) although the study was not sufficiently powered to establish superiority for individual outcomes. No significant differences were seen for other components: nonfatal MI occurred in 2.3% of patients in the oral semaglutide group and in 1.9% in the placebo group (HR 1.18; 95% CI 0.73–1.90), while nonfatal stroke occurred in 0.8% and 1.0%, respectively (HR 0.74; 95% CI 0.35–1.57). Rates of death from any cause were 1.4% with oral semaglutide and 2.8% with placebo (HR 0.51; 95% CI 0.31–0.84). An expanded composite endpoint of MACE plus unstable angina resulting in hospitalization or HF resulting in hospitalization occurred in 5.2% of the patients receiving subcutaneous semaglutide and in 6.3% of those receiving placebo (HR 0.82; 95% CI 0.61–1.10). The PIONEER 6 study investigators therefore concluded that the CV safety profile of oral semaglutide was noninferior to placebo, when both were administered with a background of standard care.

Whether oral semaglutide significantly reduces the risk of MACE is the subject of the ongoing CVOT, A Heart Disease Study of Semaglutide in Patients with Type 2 Diabetes (SOUL; NCT03914326) (13). Larger and longer than PIONEER 6, SOUL is evaluating the effects of once-daily oral semaglutide (up to 14 mg) vs. placebo in around 9,640 patients with T2D and CVD, cerebrovascular disease, symptomatic peripheral artery disease, or CKD over a period of 3.5–5 years. The primary endpoint is time to the first occurrence of MACE and the trial is powered for an assessment of superiority (vs. placebo), which is part of the prespecified statistical analysis plan. Secondary endpoints include several composite endpoints, all-cause mortality, CKD-related endpoints, major adverse limb events, and individual components of the composite outcomes. The trial size and study duration of SOUL are similar to the LEADER CVOT, which compared liraglutide with placebo as described below (5).

## Pooled Analysis of SUSTAIN and PIONEER Trials

Insights from the individual trials are complemented by a recent *post-hoc* patient-level analysis that combined data from the SUSTAIN 6 and PIONEER 6 trials, which was made possible by their similar designs (11). In terms of glycemic and body weight control, once-daily oral and once-weekly subcutaneous semaglutide display very similar actions at corresponding doses (14). When data were combined, the overall HR for MACE with semaglutide vs. placebo was 0.76 (95% CI 0.62–0.92). The HRs for each individual component of MACE were <1.0 and the upper limit of the 95% CI was <1.0 for nonfatal stroke. While these are *post-hoc* analyses, they suggest a potential for beneficial effects for semaglutide on CV outcomes regardless of the route of administration.

The effect of semaglutide on MACE was consistent across several clinically relevant subgroups, including those with established CVD and/or CKD vs. those with CV risk factors only, and in patients with and without prior MI or stroke. In patients with prior HF (NYHA class II–III), no effect of semaglutide vs. placebo on MACE was observed, although the overall incidence of prior HF was low. When considering HF hospitalization as an endpoint, the pooled analysis found no effect, with a HR of 1.03 (95% CI 0.75–1.40).

The lack of an increased CV risk in SUSTAIN 6 and PIONEER 6 is consistent with evidence from a meta-analysis summarizing data from SUSTAIN and PIONEER glycemic efficacy trials, which included patients with T2D at relatively low CV risk (11). When MACE were analyzed in the SUSTAIN 1–5 and two SUSTAIN Japanese trials and in PIONEER 1–5, 7–8 and two PIONEER Japanese trials, the pooled incidence rates were low at 0.7 and 0.9 events per 100 subject-years with semaglutide and comparator, respectively. The HR for MACE was 0.85, with broad 95% CIs (95% CI 0.55–1.33) due to the low numbers of events accrued.

## Cardiovascular Safety of Other GLP-1RAS

**Figure 2:**
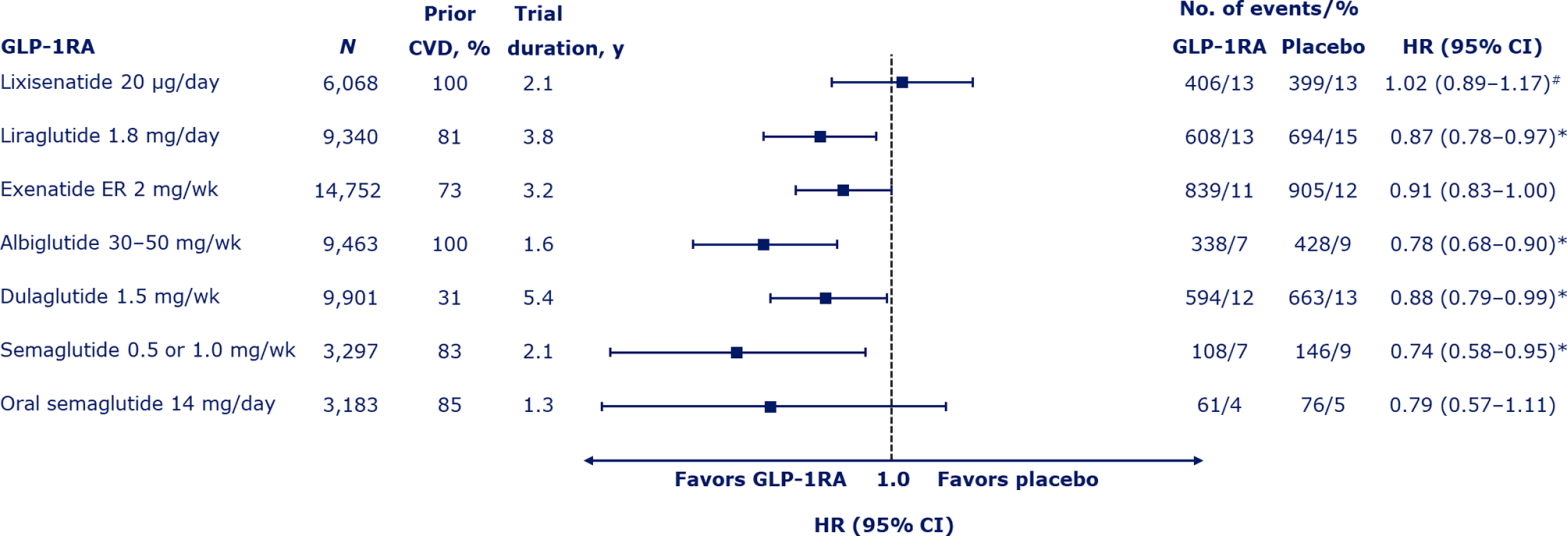
Risk of major adverse cardiovascular events (death from cardiovascular causes, nonfatal myocardial infarction, or nonfatal stroke) with GLP-1RAs (4–10). Median duration of the trials shown. ^#^Also includes hospitalization for unstable angina. *Denotes significant difference (p < 0.05) vs. placebo. CI, confidence interval; CVD, cardiovascular disease; ER, extended release; GLP-1RA, glucagon-like peptide-1 receptor agonist; HR, hazard ratio; wk, week; y, years.

To date, seven CVOTs have been conducted with GLP-1RAs (4–10) and results for effects on MACE are shown in **Figure 2**. The trials varied in their ambitions (striving for noninferiority or superiority) and therefore had different population sizes and durations. There was also some variation in the population characteristics studied.

The first CVOT with GLP-1RAs was the ELIXA trial with the short-acting agent, lixisenatide, administered by once-daily subcutaneous injection (4). In 6,068 patients who had had a recent acute coronary event (within 180 days), the primary endpoint of MACE plus hospitalization for unstable angina occurred with a HR of 1.02 (95% CI 0.89–1.17) over approximately 2 years of follow up, demonstrating noninferiority of lixisenatide to placebo (*p* < 0.001) but with no positive effects, despite adequate power for superiority testing (*p* = 0.81). There was no significant difference in the risk of all-cause mortality (0.94; 95% CI 0.78–1.13).

The next CVOT to be published, the post-approval LEADER trial with once-daily subcutaneous liraglutide, gave the first indications that GLP-1RAs could be capable of exerting CV benefits. The rate of MACE was significantly lower with liraglutide than with placebo (HR 0.87; 95% CI 0.78–0.97; *p* < 0.001 for noninferiority; *p* = 0.01 for superiority) over ~4 years in 9,340 patients with established CVD or CV risk factors (5). The risk of all-cause mortality was also lower in the liraglutide group than the placebo group (HR 0.85; 95% CI 0.74–0.97; *p* = 0.02).

The EXSCEL trial was a large trial of 14,752 patients with established CVD or CV risk factors followed for a median of 3.2 years, which studied the effects of subcutaneous once-weekly exenatide extended release (ER) (7). The HR for MACE with exenatide vs. placebo was 0.91 (95% CI 0.83–1.00), demonstrating noninferiority vs. placebo (*p* < 0.001), but not superiority (*p* = 0.06). The HR for all-cause mortality was 0.86 (95% CI 0.77–0.97), which was not considered significant based on the hierarchical testing plan.

Superiority in MACE was subsequently shown for once-weekly subcutaneous albiglutide vs. placebo in the Harmony Outcomes trial (HR 0.78; 95% CI 0.68–0.90; *p* < 0.0001 for noninferiority; *p* = 0.0006 for superiority), which studied 9,463 patients aged ≥40 years with established CVD over a median of 1.6 years (8). The HR for all-cause mortality was 0.95 (95% CI 0.79–1.16; *p* = 0.644). However, albiglutide had been withdrawn from the market due to limited prescribing prior to analyzing this CVOT and has since not been available.

Superiority was also demonstrated with once-weekly subcutaneous dulaglutide vs. placebo in the REWIND trial over a long median follow-up period of 5.4 years (HR for MACE of 0.88; 95% CI 0.79–0.99; *p* = 0.026 for superiority) (9). All-cause mortality did not differ significantly between the dulaglutide and placebo groups (HR 0.90; 95% CI 0.80–1.01; *p* = 0.067). The REWIND trial was noteworthy as, from its total of 9,901 participants, the majority (68.5%) had CV risk factors only at baseline and fewer than one-third (31.5%) had established CVD. In contrast, the prevalence of established CVD was 73–100% in other CVOTs (4–10). The authors of REWIND concluded that GLP-1RAs should be considered for the management of glycemic control in people with T2D with either previous CVD or CV risk factors.

Thus, overall, GLP-1RAs appear to have the potential for beneficial effects on adverse CV outcomes, especially concerning ischemic events and related mortality. A recent meta-analysis of the seven CVOTs indicated that GLP-1RA treatment reduced MACE by 12% (HR 0.88; 95% CI 0.82–0.94; *p* < 0.001) (15). In addition, CV mortality was reduced by 12% (HR 0.88; 95% CI 0.81–0.96; *p* = 0.003), fatal or nonfatal stroke by 16% (HR 0.84; 95% CI 0.76–0.93; *p* < 0.001), and fatal or nonfatal MI by 9% (HR 0.91; 95% CI 0.84–1.00; *p* = 0.043). Another meta-analysis of the same seven trials similarly reported that GLP-1RAs significantly reduced MACE, with a number needed to treat (NNT) to prevent one MACE of 73 (95% CI 45–212) (16). GLP-1RAs also reduced total mortality by 11%, with an NNT to prevent one death of 118, reduced CV mortality by 12% (NNT 170), and reduced stroke by 16% (NNT 211). However, they are less effective regarding hospitalization due to HF, with a reduction shown of 8% (NNT 300).

Reasons that have been postulated to account for the lack of positive effect in the ELIXA trial are the different trial populations (such that biological processes after an acute coronary event may be less amenable to modification than those relating to general atherosclerosis) and the short duration of action of lixisenatide (insufficient GLP-1 receptor stimulation over the 24-hour dosing period) (17). The effect of exenatide ER was also less positive than the other GLP-1RAs across a large population; this may be as a result of the dose of 2 mg per week, which may not be competitive compared with the doses of other GLP-1RAs (17). Also, a relatively high number of patients discontinued treatment in EXSCEL, which may have prevented determination of a treatment difference between exenatide ER and placebo (7).

## What Is the Place of GLP-1RAS in the Guidelines for Patients With Diabetes and Cardiovascular Disease?

Based on these CVOTs, the American Diabetes Association (ADA)/European Association for the Study of Diabetes (EASD) have provided updated guidance (18). They recommend that for patients with T2D and established atherosclerotic CVD (such as those with prior MI, ischemic stroke, unstable angina with electrocardiogram changes, myocardial ischemia on imaging or stress test, or revascularization of coronary, carotid, or peripheral arteries) where ‘MACE is the gravest threat’, the level of evidence for MACE benefit is greater for GLP-1RAs than other glucose-lowering classes, in particular, SGLT2is (18). To reduce the risk of MACE, the consensus update states that GLP-1RAs can also be considered in patients with T2D without established CVD but with indicators of high risk, specifically, patients aged ≥55 years with coronary, carotid, or lower extremity artery stenosis >50%, left ventricular hypertrophy, eGFR <60 mL/min/1.73 m^2^, or albuminuria.

As GLP-1RAs do not appear to have a consistent effect on HF hospitalization, SGLT2is are recommended if HF predominates; however, if SGLT2is are not tolerated or are contraindicated, or if eGFR is less than adequate, a GLP-1RA with proven CV benefit can be added (18). SGLT2is are also recommended first for patients where CKD predominates (18). Nevertheless, some beneficial effects of GLP-1RAs on albuminuria and reducing the progressive loss of kidney function have been demonstrated in LEADER, SUSTAIN 6, and REWIND (5, 6, 9, 19) and a GLP-1RA with proven CVD benefit is recommended in patients with CKD if SGLT2is are not tolerated or are contraindicated, or if eGFR is less than adequate (18).

In recent guidelines on diabetes, prediabetes, and CVD from the European Society of Cardiology, in collaboration with the EASD, GLP-1RAs with proven CV benefit (liraglutide, semaglutide, and dulaglutide) are recommended as an add-on therapy to metformin (20). GLP-1RAs with proven benefit are even recommended as a first-line therapy in people with T2D and atherosclerotic CVD or at high/very high CV risk without prescribing metformin (20), despite the fact that CVOTs testing GLP-1RAs have mainly been performed in metformin-treated patients. The high-risk category is defined by ≥10 years of (known) diabetes duration, without target organ damage, but one (or more) other associated risk factor (such as obesity, hypertension, dyslipidemia, or smoking). GLP-1RAs with proven CV benefit are recommended in patients with T2D and CVD or at very high/high CV risk to reduce CV events (class I, level A), while liraglutide is also recommended to reduce the risk of death (class I, level B) (20). Many diabetologists still favor a combination of a GLP-1RA or SGLT2i with metformin (if not contraindicated and if no intolerance precludes the use of metformin), even if these combinations do not lead to achievement of glycemic targets. More research is needed to guide first-line recommendations.

## Mechanisms for the Cardiovascular Benefits of GLP-1RAS

**Figure 3:**
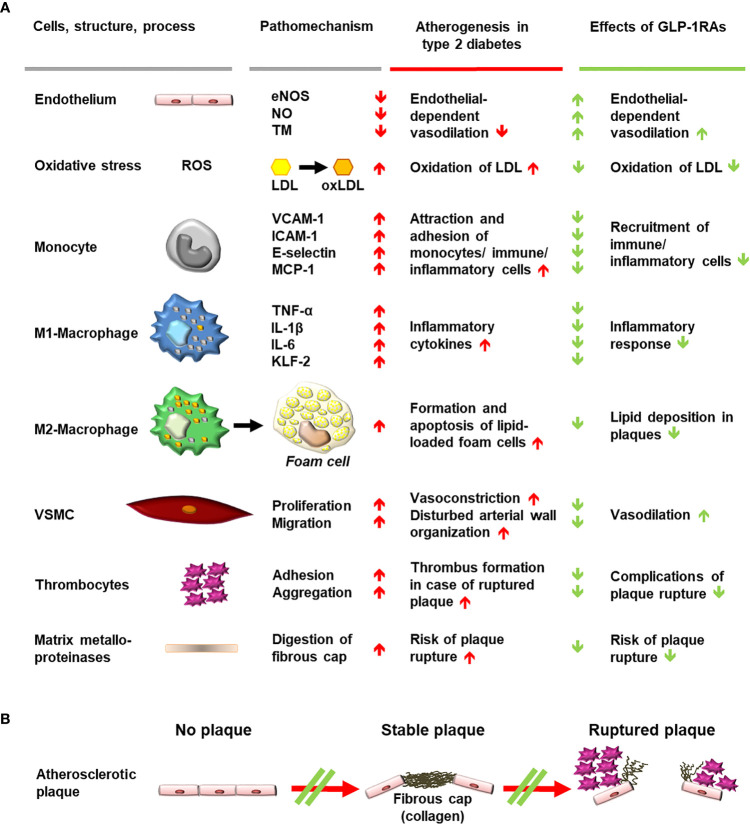
Schematic diagram of mechanisms involved in generating atherosclerotic lesions in patients with type 2 diabetes and anti-atherosclerotic effects of stimulating GLP-1 receptors with GLP-1RAs. **(A)** Cells, structures, and processes involved in atherogenesis, for which evidence suggests an interference of GLP-1 receptor stimulation with pro-atherogenic mechanisms. Findings worsening the progression of atherogenesis are depicted as red arrows, while beneficial effects of GLP-1RAs on pathomechanisms and atherogenesis are shown as green arrows. **(B)** Progression of plaque formation towards an increased likelihood of rupture is shown with red arrows. Interference with the formation of plaques or with progression towards plaque rupture is shown as a green double line crossing a red arrow. eNOS, endothelial nitrous oxide synthase; GLP-1, glucagon-like peptide-1; GLP-1RA, glucagon-like peptide-1 receptor agonist; ICAM-1, intercellular adhesion molecule 1; IL, interleukin; KLF-2, Krüppel-like factor 2; LDL, low-density lipoprotein; MCP-1, monocyte chemoattractant protein 1; NO, nitrous oxide; oxLDL, oxidized low-density lipoprotein; ROS, reactive oxygen species; TM, thrombomodulin; TNF-α, tumor necrosis factor alpha; VCAM-1, vascular cell adhesion protein 1; VSMC, vascular smooth muscle cell.

Different mechanisms have been proposed to explain the CV benefits elicited by some GLP-1RAs (21). GLP-1RAs have positive effects on several CV risk factors (glycemic control, body weight, blood pressure, fasting, and postprandial lipoproteins) and it appears that the GLP-1RAs that induced the largest reductions in HbA_1c_, body weight, and systolic blood pressure were also those associated with CV-event reduction. However, not all glucose-lowering and risk-factor management trials have shown a positive effect on CV events over the relatively short timeframe of CVOTs and therefore, risk-factor modification alone cannot explain the magnitude of the benefits observed (21). GLP-1RAs may exert additional mechanisms involving directly influencing GLP-1 receptors in the CV system, potentially leading to anti-atherosclerotic/anti-inflammatory effects and improved endothelial function/vasodilation (**Figure 3**) (21).

In atherogenesis, low-density lipoprotein (LDL) cholesterol is transported into the intima layer of arterial blood vessels where reactive oxygen species (ROS) can lead to the formation of oxidized LDL (oxLDL) particles. In an environment characterized by oxidative stress and mitochondrial dysfunction, the presence of oxLDL further increases the secretion of proinflammatory cytokines (e.g., tumor necrosis factor α, interleukin [IL]-6 and IL-1β) and the expression of adhesion molecules (including vascular cell adhesion protein [VCAM]-1, monocyte chemoattractant protein [MCP]-1, intercellular adhesion molecule [ICAM]-1 and E-selectin) by monocytes and macrophages (22). In addition, the Krüppel-like factor 2 pathway is suppressed, leading to decreased endothelial nitric oxide (NO)-synthase (eNOS) activity, reduced NO production, vasoconstriction, vascular smooth muscle cells (VSMC) proliferation, and intima-media thickening (22, 23).

GLP-1 receptor stimulation appears to attenuate these processes in preclinical models and human studies in various ways. GLP-1 receptor stimulation (e.g., through GLP-1 (24–27), exenatide (28), liraglutide (26, 29–32), or semaglutide (33) prevents ROS and reduces vascular oxidative stress. The secretion of adhesion molecules including VCAM-1, MCP-1, ICAM-1, and E-selectin is also reduced by GLP-1 (34), exenatide (34–36), liraglutide (37), and dulaglutide (22). Furthermore, increased eNOS activity and NO production have been observed with GLP-1 (23, 26), exenatide (23), and liraglutide (26, 30, 37). In addition, liraglutide has been reported to reduce oxLDL uptake into macrophages (38), inhibit VSMC proliferation (39), and reduce carotid intima-media thickness in patients with the metabolic syndrome (40).

GLP-1RAs [lixisenatide (41) and liraglutide (42)] may also modulate the ROS- and oxLDL-mediated differentiation of macrophage phenotype away from the inflammatory pattern of M1-macrophages and towards anti-inflammatory M2-macrophages. Furthermore, GLP-1 (43, 44) and liraglutide (45) may suppress oxLDL-induced foam-cell formation from M2-macrophages, retarding atherosclerotic lesion development in experimental models (45). Evidence suggests that GLP-1 (46) and lixisenatide (47) may also stabilize atherosclerotic plaques, reducing plaque macrophage infiltration, increasing collagen content, and increasing fibrous cap thickness. In addition, the activity of matrix metalloproteinases, which destabilize the dense fibrous cap of stable plaques through proteolysis, is reduced by GLP-1RAs [GLP-1 (46), exenatide (48) or semaglutide (49)]. Plaque hemorrhage is reduced by semaglutide (49), which, like GLP-1 (50), also inhibits caspase-mediated apoptosis (51). The integrity of endothelial cells was shown to be stabilized by exenatide, suggesting further protective effects of GLP-1 receptor stimulation (52, 53).

## Future Clinical Trials Examining Semaglutide Effects on Cardiovascular Disease

To provide further insight, the effect of subcutaneous semaglutide vs. placebo on coronary atherosclerosis progression is currently being measured by multidetector computed tomography angiography over 1 year in ~140 patients with T2D and CVD or at least one CV risk factor in the Semaglutide Treatment On Coronary Progression (STOP; NCT03985384) trial (54, 55). Secondary endpoints include quantitative changes in different coronary plaque types and morphology. In addition, the LIRA-FLAME trial (NCT03449654) is examining the effects of liraglutide vs. placebo on vascular inflammation in 102 patients with T2D over 26 weeks as assessed by fluorodeoxyglucose-positron emission tomography/computed tomography (primary outcome), and also to evaluate endothelial function, coronary artery calcium, and carotid-intima thickness (56).

## Conclusions

The CV safety of semaglutide, administered subcutaneously or orally, has been established in the SUSTAIN 6 and PIONEER 6 trials. These findings are consistent with the results of CVOTs conducted for different GLP-1RAs. The beneficial effects of liraglutide, semaglutide, and dulaglutide have been recognized in international guidelines and these GLP-1RAs are now recommended to reduce the risk of CV events in high-risk patients. The ongoing SOUL trial will confirm whether oral semaglutide provides significant reductions in CV events as seen with subcutaneous semaglutide. The SELECT trial will assess whether subcutaneous semaglutide improves CV outcomes in obese or overweight patients without T2D. A CV benefit in this trial may indicate the need for earlier intervention in CV risk reduction, even before the development of T2D. The mechanisms responsible for the reduced risk of adverse CV events with GLP-1RAs may be related to inhibition of the progression of atherosclerotic lesions by multiple pathways, primarily involving reduced inflammatory processes within the atherosclerotic plaque. Additional studies are warranted, and ongoing studies will provide further mechanistic information into how some GLP-1RAs are able to provide CV benefits.

## Author Contributions

All authors contributed to the article and approved the submitted version.

## Funding

This article was supported by Novo Nordisk, who was provided with the opportunity to perform a medical accuracy review.

## Conflict of Interest

MN has been a member of advisory boards or has consulted with AstraZeneca, Boehringer Ingelheim, Eli Lilly & Co., Fractyl, GlaxoSmithKline, Menarini/Berlin Chemie, Merck, Sharp & Dohme, and Novo Nordisk. He has received grant support from AstraZeneca, Eli Lilly & Co., Menarini/Berlin-Chemie, Merck, Sharp & Dohme, Novartis Pharma, and Novo Nordisk. He has also served on the speakers’ bureau of AstraZeneca, Boehringer Ingelheim, Eli Lilly & Co., Menarini/Berlin Chemie, Merck, Sharp & Dohme, Novo Nordisk, and Sun Pharma.

The remaining author declares that the research was conducted in the absence of any commercial or financial relationships that could be construed as a potential conflict of interest.

## Abbreviations

CI, confidence interval; CKD, chronic kidney disease; CV, cardiovascular; CVD, cardiovascular disease; CVOT, cardiovascular outcomes trial; EASD, European Association for the Study of Diabetes; eGFR, estimated glomerular filtration rate; eNOS, endothelial nitric oxide synthase; ER, extended release; GLP-1, glucagon-like peptide-1; GLP-1RA, glucagon-like peptide-1 receptor agonist; HbA_1c_, glycated haemoglobin; HF, heart failure; HR, hazard ratio; ICAM, intercellular adhesion molecule; IL, interleukin; KLF-2, Krüppel-like factor 2; LDL, low-density lipoprotein; MACE, major adverse cardiovascular event(s); MCP, monocyte chemoattractant protein; MI, myocardial infarction; NNT, number needed to treat; NO, nitric oxide; NYHA, New York Heart Association; oxLDL, oxidized low-density lipoprotein; PIONEER, Peptide InnOvatioN for Early diabEtes tReatment; ROS, reactive oxygen species; SGLT2i, sodium-glucose co-transporter-2 inhibitor; SOUL, A Heart Disease Study of Semaglutide in Patients with Type 2 Diabetes; SUSTAIN, Semaglutide Unabated Sustainability in Treatment of Type 2 Diabetes; T2D, type 2 diabetes; TM, thrombomodulin; TNF-α, tumor necrosis factor alpha; VCAM, vascular cell adhesion protein; VSMC, vascular smooth muscle cells.
